# Auxotonic to isometric contraction transitioning in a beating heart causes myosin step-size to down shift

**DOI:** 10.1371/journal.pone.0174690

**Published:** 2017-04-19

**Authors:** Thomas P. Burghardt, Xiaojing Sun, Yihua Wang, Katalin Ajtai

**Affiliations:** 1 Department of Biochemistry and Molecular Biology, Mayo Clinic Rochester, Rochester, Minnesota, United States of America; 2 Department of Physiology and Biomedical Engineering, Mayo Clinic Rochester, Rochester, Minnesota, United States of America; Semmelweis Egyetem, HUNGARY

## Abstract

Myosin motors in cardiac ventriculum convert ATP free energy to the work of moving blood volume under pressure. The actin bound motor cyclically rotates its lever-arm/light-chain complex linking motor generated torque to the myosin filament backbone and translating actin against resisting force. Previous research showed that the unloaded in vitro motor is described with high precision by single molecule mechanical characteristics including unitary step-sizes of approximately 3, 5, and 8 nm and their relative step-frequencies of approximately 13, 50, and 37%. The 3 and 8 nm unitary step-sizes are dependent on myosin essential light chain (ELC) N-terminus actin binding. Step-size and step-frequency quantitation specifies in vitro motor function including duty-ratio, power, and strain sensitivity metrics. In vivo, motors integrated into the muscle sarcomere form the more complex and hierarchically functioning muscle machine. The goal of the research reported here is to measure single myosin step-size and step-frequency in vivo to assess how tissue integration impacts motor function.

A photoactivatable GFP tags the ventriculum myosin lever-arm/light-chain complex in the beating heart of a live zebrafish embryo. Detected single GFP emission reports time-resolved myosin lever-arm orientation interpreted as step-size and step-frequency providing single myosin mechanical characteristics over the active cycle. Following step-frequency of cardiac ventriculum myosin transitioning from low to high force in relaxed to auxotonic to isometric contraction phases indicates that the imposition of resisting force during contraction causes the motor to down-shift to the 3 nm step-size accounting for >80% of all the steps in the near-isometric phase. At peak force, the ATP initiated actomyosin dissociation is the predominant strain inhibited transition in the native myosin contraction cycle. The proposed model for motor down-shifting and strain sensing involves ELC N-terminus actin binding.

Overall, the approach is a unique bottom-up single molecule mechanical characterization of a hierarchically functional native muscle myosin.

## Introduction

The myosin motor protein powers the beating heart with transduction of ATP to mechanical work. Rationalizing “bottom-up” single myosin mechanics with “top-down” whole animal muscle physiology is indispensable to solving myosin’s structure/function paradigm for creating an ensemble capable nanomotor and to providing the insight into muscle disease mechanisms demanded by translational science. A time-resolved in vivo imaging approach characterizes single myosin mechanics in contracting striated muscle of live zebrafish embryos. It provides the means for linking bottom-up myosin characteristics to top-down muscle physiology or phenotype in the zebrafish embryo model for human muscle.

The myosin transducer has a globular head (subfragment 1 or S1) and tail domain that forms myosin dimers and assembles into thick filaments. Thick filaments interdigitate with actin thin filaments in striated muscle and slide relatively during contraction [1]. S1 contains ATP and actin binding sites and a swinging lever-arm that cyclically applies tension to power filament sliding while myosin is strongly actin bound. The lever-arm converts torque generated in the motor into linear displacement (step-size) and undergoes strain due to the resisting force. Strain affects the lever-arm and the bound essential and regulatory light chains (ELC and RLC). RLC stabilizes the lever-arm [2] and disease implicated RLC mutants lower velocity, force, and strain sensitivity suggesting they alter lever-arm processing of stress [3]. The ELC N-terminus binds actin to modulate myosin functionality [4] and step-size in cardiac muscle [5].

In published work, we tagged human ventricular cardiac RLC (MYL2) at the C-terminus with green fluorescent protein (HCRLC-GFP) then exchanged the chimer into permeabilized skeletal [6] or cardiac papillary muscle fibers [7]. Extensively and specifically exchanged myosin in these fibers supports native isometric contraction implying the GFP tag does not affect muscle contraction. The photoactivatable variant, HCRLC-PAGFP, was individually activated in the exchanged papillary muscle fibers isolating single myosins *in situ*. Super-resolved orientation of single myosin lever-arms was measured from fibers in rigor, relaxation, and active isometric conditions. Single molecule orientation was also measured for the exchanged HCRLC-PAGFP modified by disease linked mutations to the HCRLC [3]. The exchanged mutant HCRLC lowered lever-arm stiffness and impaired lever-arm transduction/mechanical-coupling.

The zebrafish embryo is transparent to visible light allowing deep imaging using wide field fluorescence microscopy with highly inclined (HILO) illumination [8]. In the present work, new transgenic zebrafish were created by inserting the HCRLC-GFP or HCRLC-PAGFP gene into the zebrafish genome and using the cmlc2 promoter to drive gene expression in the heart [9]. Transgenic zebrafish embryos had visible GFP expression confined to the heart ventricle and arranged in the striated pattern characteristic to myosin in cardiac muscle fiber sarcomeres indicating specific myosin binding. Embryonic and adult transgenic zebrafish have normal heart phenotype and function. At 3–4 days post fertilization (dpf), embryos expressing HCRLC-PAGFP were imaged using HILO to detect single cardiac myosin lever-arms in the beating heart. The native embryonic heart runs with ~120 beats per min (bpm) at room temperature. We slowed heart rate to ~60 bpm using a reversible anesthetic treatment affecting nervous controlled cardiac pacing and imaged single cardiac myosins at 10 frames per second. Usually one heart cycle was quantitated in 10 sequential images. Each image acquired light for slightly less than 100 ms. This sampling rate captured and quantitated interesting mechanical features of the single functioning cardiac myosins while faster frame rates failed to provide an adequate signal to noise (S/N) ratio profile for quantitation. Control imaging experiments were also conducted on relaxed hearts temporarily stopped by a higher dose anesthetic treatment. The heart contraction cycle separated into late relaxed (diastole and full heart), auxotonic active (early systole where cardiac force exceeds load), and isometric active (late systole before ejection where cardiac force equals load) phases with distinct myosin mechanical characteristics. Comparison of single myosin mechanics in auxotonic and isometric contraction suggests a new paradigm for mechanical regulation of force/velocity.

Myosin mechanical functionality in vitro and at the single molecule level precisely characterizes the essential myosin structure/function paradigm but in the absence of the other motors and proteins in the muscle sarcomere. The native myosin is more complex with potential for hierarchical coordinated functionality because of its structured environment. The zebrafish embryo model system provides opportunity for single molecule mechanical characterization of a naturally integrated cardiac myosin. We measure in vivo cardiac myosin lever-arm rotation and interpret data as the step-size and step-frequency. These metrics uniquely specify native myosin functionality for an unprecedented view into how tissue integration shapes motor function.

## Materials and methods

### A. Zebrafish sample preparations

#### Ethics

Zebrafish embryos were produced and used in this study with approval from the Mayo Clinic Rochester Institutional Animal Care and Use Committee (protocol A47113).

#### Zebrafish adult ventricular cardiac myosin extraction and preparation

2–6 adult wild type (WT or Zmys) 11 month old dominant leopa zebrafish were obtained from the Mayo Clinic Zebrafish Core Facility. Fish were anesthetized for 3–5 minutes using tricaine methanesulfonate (MS-222, 168 mg/L) then euthanized. Hearts were dissected by using forceps and the ventricle placed into 1.5 ml eppendorf tubes containing 40 μL/(5 hearts) SDS sample buffer (0.125 M Tris-HCL pH 6.8, 10% glycerol, 2% SDS, 5% β-mercaptoethanol, and 0.5 mg/ml bromophenol blue). The tubes were vortexed, pelleted in the centrifuge, and heated to 95°C for 5 minutes. The pellet was homogenized by hand using a pellet pestle then incubated at room temperature for 30 min. The supernatant containing the extracted and denatured cardiac tissue proteins were heated again to 95°C for 5 minutes and loaded to a gel or stored in the freezer at -20°C.

#### Zebrafish embryo cardiac myosin extraction

Dpf 6 embryos (~80) were anesthetized for 3–5 minutes using tricaine then euthanized. Embryos were transfer into E3 water (5 mM NaCl, 0.17 mM KCl, 0.33 mM CaCl_2_, 0.33 mM MgSO_4_, 10^−5^% methylene blue) onto a 16-well glass slide. The hearts were dissected under the microscope using two insulin needles and excess buffer removed. We added 2–3 μL 3% SDS sample buffer (without bromophenol blue) to each well then transferred the tissue to a 1.5 ml Eppendorf tube on ice. The tissue sample was centrifuged at low speed, denatured at 95°C for 5 min, sonicated for 2 min, incubated at room temperature for 30 min and then centrifuged with 14000g for 10 min at 4°C. Supernatant containing solubilized protein was concentrated using ice cold 20% trichloro acetic acid (TCA) precipitation, vortexed and incubated for 30 minutes on ice, centrifuged for 30 minutes at top speed in a microcentrifuge at 4°C then the supernatant was carefully removed to avoid disturbing the pellet. We added 0.5 ml cold acetone to the pellet to remove traces of TCA, vortexed briefly, centrifuged for 15 minutes at top speed in microcentrifuge at 4°C, removed supernatant and dried the pellet with nitrogen gas. Dried pellet was resuspended in 2% SDS sample buffer with bromophenol blue, boiled, centrifuged at low speed to remove particulate matter, then loaded on the gel or stored at -20°C.

#### Transgenic animals expressing HCRLC-GFP or HCRLC-PAGFP

We coinjected transposase mRNA and plasmid containing a Tol2 construct with the zebrafish cmlc2 enhancer and the gene for HCRLC-GFP or HCRLC-PAGFP into the cytoplasm of one-cell-stage embryos. Cmlc2 enhancer drives HCRLC-(PA)GFP expression specifically in cardiac muscle [9]. At 3–4 dpf, we screened embryos for green fluorescence excited by 405 nm transmitted light using a 10X objective. Selected embryos exhibiting (PA)GFP expression were raised to produce F0 adults according to standard rearing protocols in the zebrafish core facility. After three months, the founder fish (F0) were screened by outcrossing with wild type. The F1 embryo offspring were used for experiments or grown to maturity for an F2 generation.

Transgenic zebrafish had visible (PA)GFP expression confined to the heart and in the striated pattern similar to that in skeletal muscle of transiently expressed HCRLC-(PA)GFP using the UNC-45b enhancer [10, 11]. Wild type fish were incrossed as control and F1 transgenic fish were outcrossed with wild type producing F2 embryos. F2 transgenic GFP(+) or GFP(−) (TgGFP(+) or TgGFP(−)) embryos having strong or weak (PA)GFP expression in the heart were selected at 3–4 dpf. Transgenic animals are indicated by TgGFP for either GFP or PAGFP chromophores. Single myosin imaging is always performed with the PAGFP containing isoform.

Embryos were usually treated with 0.5 mM 1-phenyl 2-thiourea (PTU) at 1 dpf to inhibit melanogenesis. Experiments were conducted at room temperature (20–22°C).

#### Cardiac functionality in zebrafish embryos

Zebrafish embryo cardiac function was estimated at 3 dpf using an ellipsoid of revolution to approximate ventricle shape with long (*a*) and short (*b*) axes measured from movies of the functioning hearts [12]. Cardiac performance was described by 4 parameters consisting of heart rate, ventricle shortening fraction (SF), ventricle end systolic (smallest) volume, and ventricle end diastolic (largest) volume. The shortening fraction measures the maximum change in *a* relative to the longest *a* over a contraction cycle. Measurements were conducted on WT and transgenic embryos with and without PTU treatment.

#### Quantitation of HCRLC-(PA)GFP tagged myosin in zebrafish embryos

Native zebrafish cardiac ventricular regulatory light chain ZRLC and HCRLC-(PA)GFP protein expression levels were measured using SDS-PAGE of expressed and extracted proteins. Quantities of purified embryonic myosin were too low to detect quantitatively on a Sypro Ruby stained gel hence we combined Sypro Ruby staining and protein immunoblotting. Purified native adult zebrafish ventricular cardiac myosin and an *in vitro* expressed HCRLC standard were run on adjacent lanes on an SDS-PAGE gel that was stained with Sypro Ruby. Intensity for the ZRLC and HCRLC bands were compared to estimate ZRLC quantity. Protein bands from an identical gel were transferred to a membrane for Western blotting using a primary RLC antibody (1:1000 dilution, product number 10906-1-AP, Proteintech, Chicago, IL)[13]. Antibody staining of the known amounts of ZRLC and HCRLC validated and calibrated the standardized immunoblotting protocol for their detection.

Myosin extracted from WT, TgGFP(−), and TgGFP(+) embryos were run on adjacent lanes on an SDS-PAGE gel then the proteins were transferred to a membrane for Western blotting using the RLC antibody. Intensities of the HCRLC-(PA)GFP and ZRLC bands were converted to HCRLC-(PA)GFP and ZRLC amounts and combined into the replaced fraction, ZRLC_rep_, given by,

$${ZRLC}_{rep}=\frac{\left[HCRLC-GFP\right]}{\left[HCRLC-GFP]+[ZRLC\right]}$$ (1)

where […] indicates mole protein and all bands came from the TgGFP(+) embryos. Independently, the amount of ZRLC removed was measured relative to a β-actin loading standard in WT and transgenic embryos and using the RLC and a β-actin antibody (1:2000 dilution, product number 4967, Cell Signaling Technology, Danvers, MA,) [14]. The ZRLC fraction lost due to transgenesis, ZRLC_rem_, is given by,

$${ZRLC}_{rem}=\frac{{\left[ZRLC\right]}_{\text{WT}}-{\left[ZRLC\right]}_{\text{TgGFP}(+)}}{{\left[ZRLC\right]}_{\text{WT}}}$$ (2)

Experiments were conducted at room temperature (20–22°C).

#### Single cardiac myosin imaging in a beating heart

At 3–4 dpf, TgGFP(+) embryos were imaged to detect single cardiac myosin dynamics in the ventriculum. A TgGFP(+) embryo was placed in a 200 μm deep microfluidic channel constructed from polydimethylsiloxane (PDMS) with the channel side up and immersed in 20 μL of 30% Danieau Buffer (D-buffer, 17.4 mM NaCl, 0.21 mM KCl, 0.12 mM MgSO_4_, 0.18 mM Ca(NO_3_)_2_, 1.5 mM HEPES, pH 7.6). We introduced stepwise concentrations of tricaine, up to 2000 mg/L, to reduce heart rate from 108–125 bpm to 50–60 bpm at 20°C to facilitate single myosin imaging. Heart rate returned to normal immediately after flushing out the drug with fresh buffer and the drug cleared naturally from the embryo at ≥10 minutes. Heart rate was monitored in a stereo microscope at high magnification. When heart rate stabilized the bathing solution volume was drawn down to <6 μL and the microfluidic was inverted then placed on top of a #0 glass coverslip forming a water tight seal with the glass for imaging exactly as described [10]. Embryos were imaged over time at a 10 Hz frame rate to collect single molecule emission images from photoactivated RLC-PAGFP. Imaging sessions were completed within 10 min. Heart rate was measured visually in the microscope every 2–3 min and remained constant at 50–60 bpm over the course of the experiment. Blood circulation in capillaries in the fish tail was also monitored every 2–3 min in the microscope by visualizing blood cell movement. Blood cell flow did not qualitatively change over the course of the experiment.

We briefly stopped the heart by raising tricaine to 2200 mg/l in the microfluidic containing the embryo then imaged the constantly-relaxed cardiac myosin.

### B. *In vivo* single myosin orientation quantitation

#### S1/GFP coordination and coordinates

**Fig 1:**
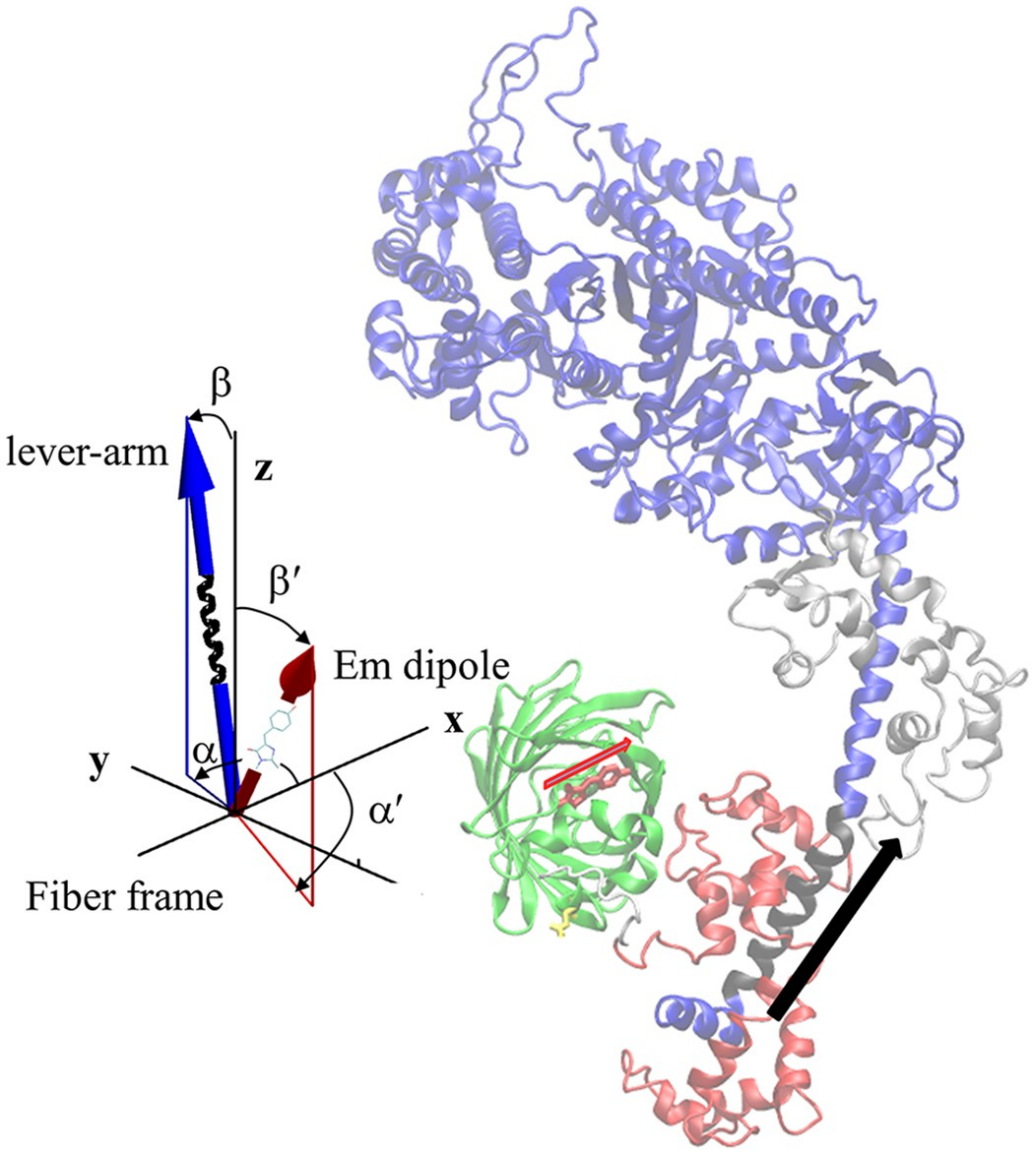
The coordination of the GFP moiety (green) and its emission dipole moment (red arrow) with myosin S1 consisting of a heavy chain (blue and black), ELC (silver), and RLC (red). The portion of the lever-arm in the heavy chain appearing in black is the α-helix segment associated with the lever-arm orientation and depicted by the black arrow. The insert shows the time-resolved coordinates for GFP chromophore emission dipole moment in red and lever-arm helix in blue corresponding to spherical polar angles (β’,α’) and (β,α) defined relative to a fiber fixed frame.

The ribbon structure in Fig 1 shows the myosin heavy chain (MHC) in blue and black, RLC and ELC binding the lever-arm in red and silver, and the GFP moiety in green. GFP is linked to the RLC C-terminus by the white linker. The S1 structure represents human β-cardiac myosin from homology modeling [15] of its sequence using the chicken skeletal myosin S1 crystal structure 2mys [16]. The arrow indicates direction of the black section of the lever-arm α-helix symmetry axis where RLC binds. The GFP chromophore in the middle of the β-barrel is indicated in red. The red arrow at the chromophore is the emission dipole orientation.

The insert has a blue arrow with embedded black α-helix representing the lever-arm with coordinates (β,α) defined relative to the unique fiber symmetry axis (z) and arbitrary x-axis defining the fiber frame. The red arrow with embedded chromophore moiety represents the GFP chromophore emission dipole related to the lab frame by (β’,α’) and referred to as probe coordinates.

**Fig 2:**
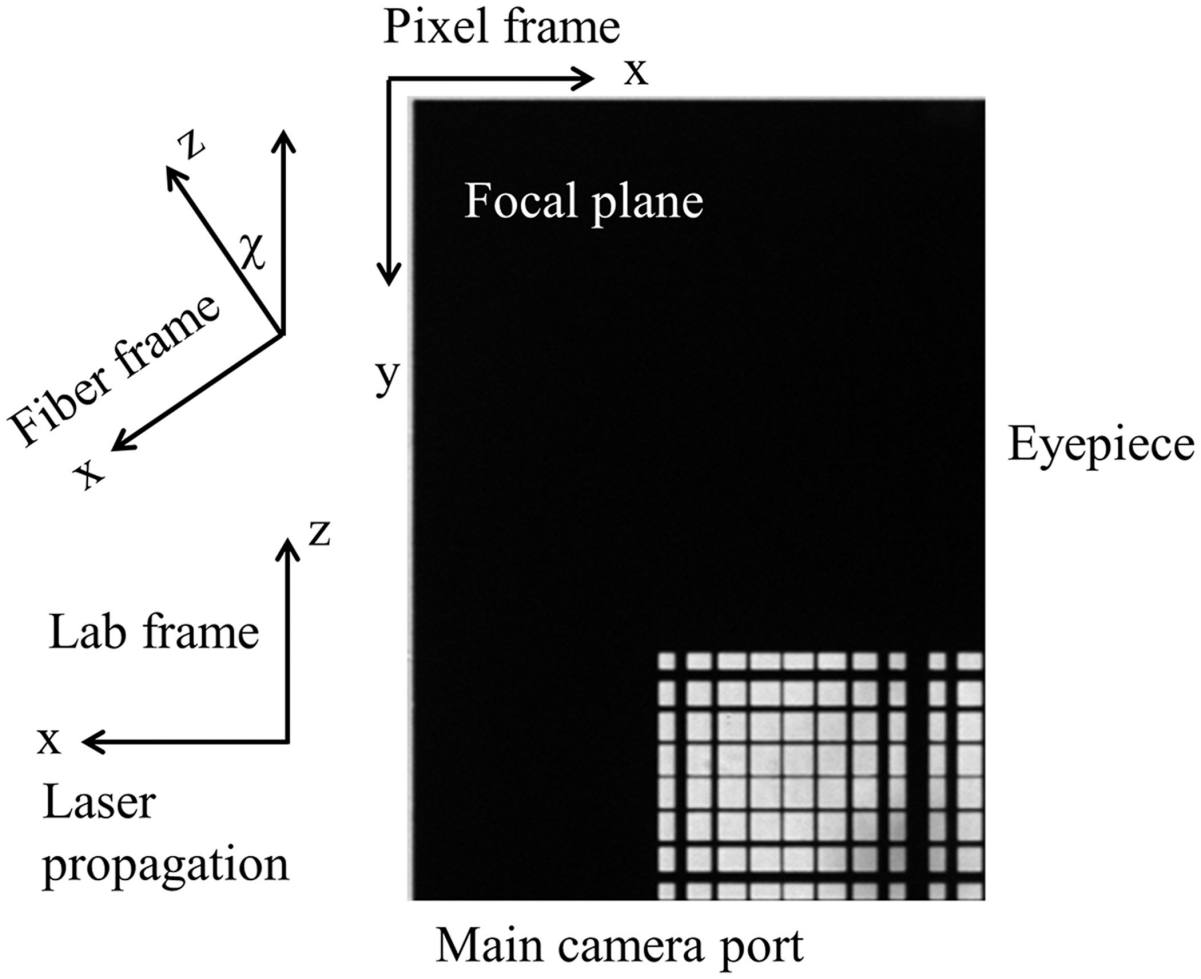
The fiber frame xz plane in Fig 1 lies in the microscope focal plane and in the lab frame as shown. Lab and fiber frames are related to each other by a rotation through χ. Images of the heart shown subsequently relate to the lab frame like the image of the test pattern.

The fiber frame xz plane in Fig 1 lies in the microscope focal plane and in the lab frame as shown in Fig 2. Lab and fiber frames are related to each other by a rotation through χ about the y-axis (y-axis not shown). Images of the heart shown in Results relate to the lab frame like the test pattern in Fig 2.

#### Orientation super-resolution measured from tagged zebrafish muscle

Zebrafish embryos confined to the microfluidic chamber were imaged with fluorescence from the cardiac tissue as described above. Single molecule fluorescence measurements from the photoactivated HCRLC-PAGFP tagged myosin lever-arms were made on an inverted microscope using highly inclined thin illumination (HILO) excitation exactly as described [10].

In all fluorescence experiments, pump and observation exciting laser light polarization is p-polarized and propagating perpendicular to the long dimension of the embryo. In the heart tissue the fiber symmetry axis is not fixed but redefined for each single myosin. A sparse population of probes is photoactivated to achieve the most selective orientation distribution of photoactivated probes by using the lowest practical pump beam intensity. We identified single molecule events by their quantized intensity change due to photoactivation or photobleaching over time. Orientation super-resolution of unit vector μ_e_[A], the *emission* dipole moment of the activated single molecule, is determined by pattern recognition exactly as described [10].

Raw GFP fluorescence intensity vs time from RLC-PAGFP tagged myosin *in vivo* from beating and relaxed zebrafish embryo heart ventriculum under HILO illumination is shown in Figs A& B in S1 File. Single myosins are identified by their quantized intensity change of ~1000 photons per 0.1 second above background due to photoactivation and subsequent photobleaching to background. Photon counts indicate intensity integrated over the EMCCD camera 11x11 pixel array containing the photoactivated chromophore image. Video files for these single myosin instances are in S1–S4 Movies.

#### S1/GFP coordination

The S1/GFP coordination of the zebrafish skeletal muscle was determined as described [10] and is shown in Fig 1. For the present study and using the same method, we checked the new dipole orientation data for relaxed and contracting muscle (summarized subsequently in RESULTS) for consistency with the S1/GFP coordination in Fig 1. We found that the new data is consistent with the previous data in selecting the S1/GFP coordination in Fig 1 over other docked models.

#### In vivo step-size

Single molecule images provided super-resolved orientation of the photoactivated RLC-PAGFP emission dipole in zebrafish cardiac muscle. Dipole orientations were computed from images that were continuously recorded for 20–40 sec. Object spatial drift, if present, was removed by large frame image alignment prior to analysis. The time-resolved coordinates have dipole or lever-arm helix spherical polar angles (β’,α’) or (β,α) defined relative to a fiber fixed frame shown in the insert to Fig 1. They are the trajectories for a single dipole or lever-arm helix.

**Fig 3:**
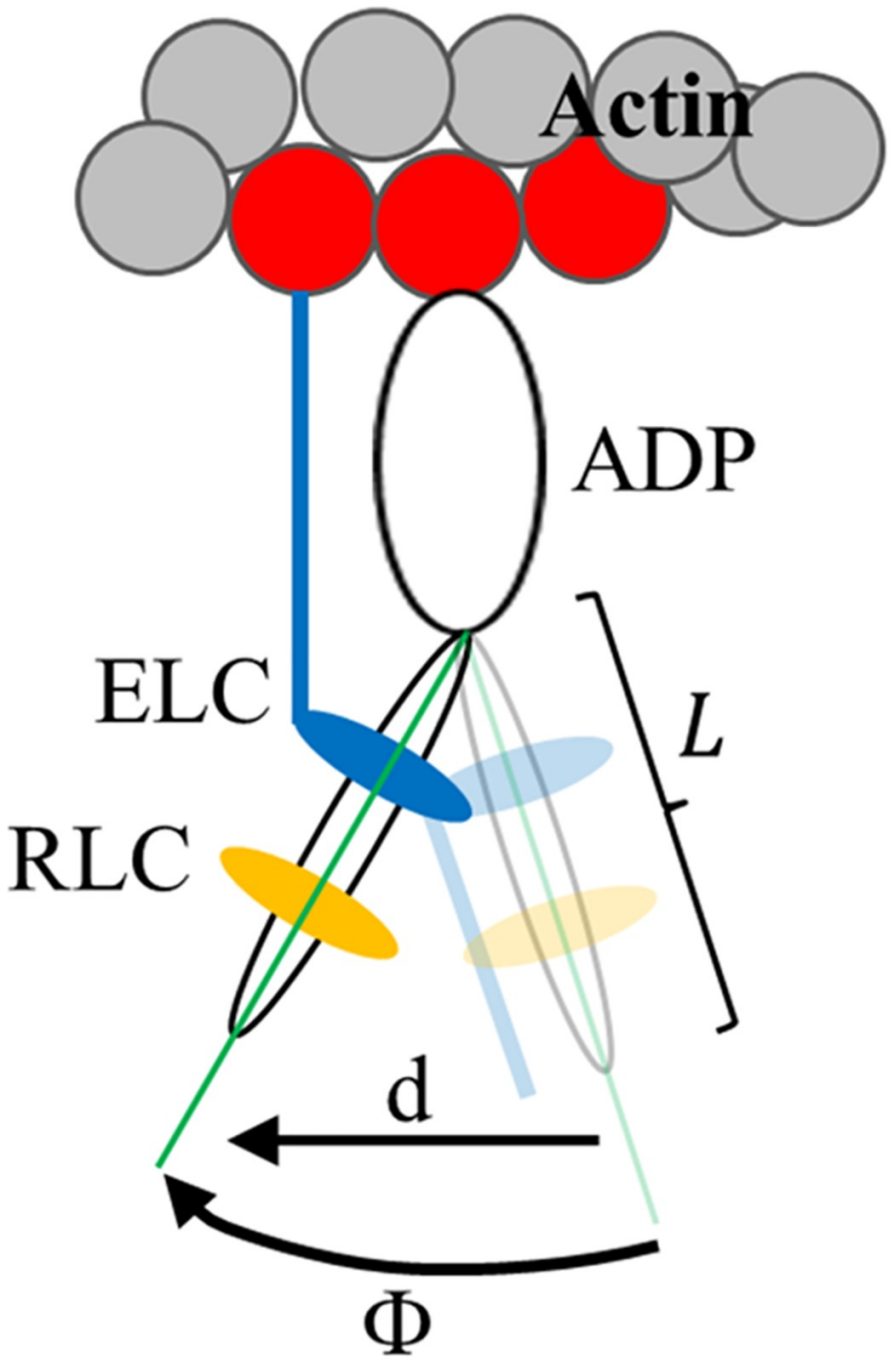
Myosin S1 consisting of a heavy chain, ELC (blue), and RLC (yellow) performing a powerstroke with a lever-arm rotation while strongly actin bound. The arc subtended by, Φ, the angle a single lever-arm helix rotates in sequential images defines a sequence of chords on a circle of diameter *L* equal to the lever-arm length indicated. Chord length is step-size, *d*, given by Eq 3. The ELC N-terminal extension, shown actin bound at the end of the powerstroke, plays a major role in the proposed mechanism for strain sensing in the myosin.

The arc subtended by, Φ, the angle a single lever-arm helix rotates in sequential time-correlated images defines a sequence of chords on a circle of diameter *L* equal to the lever-arm length indicated in Fig 3. Chord length is step-size, *d*, given by,

$$d=2L\;Sin(\frac{1}{2}\Phi )$$ (3)

Step-sizes computed by Eq 3 from many single myosin trajectories are summarized as a histogram of incremental step-lengths vs the number of observed events. Step-lengths distribute differently for various muscle physiological states. Cardiac muscle contraction separates into auxotonic and isometric phases depending on position in the heart beat cycle with the auxotonic phase early in systole when fibers are shortening but overall muscle position is relatively steady. Towards the end of systole, fiber shortening slows as force reaches isometric level and the muscle image sharpens to its peak S/N ratio.

During contraction muscle has single myosins cycling through lever-arm orientations for actin-disassociated (relaxed) and actin-associated (force generating) conformations. The relaxation phase in diastole is not identified in the beating heart cycle because the muscle translates sufficiently to leave focus. The relaxed phase is observed separately in the heart when its beat cycle is briefly interrupted by additional tricaine. Relaxed phase muscle has single myosins maintaining a mostly actin-dissociated form with statically and dynamically averaged lever-arm orientation that is non-stationary in its time sequence over sampling intervals of 100 ms. Myosin develops force with its lever-arm swing starting in the high free-energy actin bound state of the myosin most closely associated with auxotonic phase. Peak force has myosin strongly actin-bound either with ADP or after ADP release (strained rigor) most closely associated with isometric phase. Both force generating phases also contain contributions from detached (relaxed) myosins and their active counterpart (isometric with auxotonic phase or auxotonic with isometric phase) due to asynchronous myosin cycling.

We model the step-size distribution in auxotonic and isometric phases of the heartbeat, v_au_ and v_is_, with linear combination of distributions composed of relaxed, v_re_, and alternatively v_is_ and Δv_au_ for the auxotonic case or v_au_ and Δv_is_ for the isometric case, where Δv_au_ and Δv_is_ are unknown force developing homogeneous auxotonic and isometric phases of the heartbeat, subject to constraints,

$$\begin{array}{ll} \Delta {\text{v}}_{\text{au}} & ={\text{v}}_{\text{au}}-c_{1}\;{\text{v}}_{\text{is}}-c_{2}\;{\text{v}}_{re}\\ \Delta {\text{v}}_{\text{is}} & ={\text{v}}_{\text{is}}-c_{3}\;{\text{v}}_{\text{au}}-c_{4}\;{\text{v}}_{re} \end{array}$$ (4)

for unknown constants c_j_, j = 1, 2, 3, and 4. The relaxed and active step-size histograms, v_re_, v_is_, and v_au_ are the basis vectors covering probability space spanned by tagged lever-arm orientations. They have Poisson distributed noise randomly sampled while Δv_is_ and Δv_au_ are independently minimized for each trial by selection of c_j_ subject to constraints Δv_au_, Δv_is_, and c_j_ all ≥ 0 using constrained linear programing in Mathematica. We estimate the mean and variance for Δv_is_ and Δv_au_ at each point in the histogram from the family of residuals produced in the trials.

#### Pathway networks

Qdot assaying of porcine β-ventricular myosin (βmys) indicated three unitary steps-sizes of ~3, ~5, and ~8 nm with relative step frequencies of ~13, 50, and 37% [17]. Similar results were obtained using the assay for adult zebrafish skeletal myosin step-size and step-frequency [11]. We proposed that the major 5 nm step is the default step identical to the dominant step in skeletal myosin [18], that the 8 nm step is somewhat less likely and different from the 5 nm step by involving an extra interaction with actin via the unique N-terminus extension of ELC [4, 19–21], and that the minor 3 nm step is the unlikely conversion of the 5 nm step to the full cELC bound 8 nm step. The 3 nm step is isolated in time from the 5 nm step by slow ADP dissociation hence we sometimes observe it as a separate step [22]. We tested the N-terminus of ELC for its ability to regulate step-size and/or step-frequency using βmys and mouse cardiac myosin with the α heavy chain (αmys) including: a 17 residue N-terminal truncated ELC in porcine ventricular myosin made by papain digestion [23] and a 43 residue N-terminal truncated human ELC expressed in a transgenic mouse heart [24]. ELC N-terminus truncation caused significant redistribution in the step-frequencies among the unitary steps compared to control. An ensemble containing mainly myosin with ELC missing its N-terminus had significantly lower probability for making the 8 nm step but higher probability for the 5 nm step in both porcine βmys mouse αmys. ELC N-terminus truncation had little effect on the unitary myosin step-sizes [5].

The earlier in vitro work was described by a model where the 3 nm step happened only after a 5 nm step occurred. Three pathways producing 5, 8, or 5+3 nm steps describe this 3-pathway network. The present in vivo work implies that the 3 nm step can also occur independently from the 5 nm step because we observe a 3 nm step-frequency that sometimes exceeds that for 5 nm step. Four pathways producing 5, 8, 5+3, and 3 nm steps describe the new 4-pathway network that is the basis for the new model discussed in RESULTS. The 4-pathway network is a superset of the 3-pathway network.

#### Relaxed myosin lever-arm orientation dynamics

Relaxed myosin dimers in the cardiac thick filament form intra- and inter-dimer interactions thought to impact the in vivo muscle. Isolated myosin dimers exhibit the intra-dimer blocked and free head motif [25] within the hierarchical quasihelical thick filament structure [26]. The latter grants inter-dimer interactions stabilizing a globally regular but locally variable myosin 3D structure. Static variability is probably accompanied by dynamic dispersion as myosin monomers sample conformation space. We hypothesize that single myosin head dynamics observed in the relaxed zebrafish cardiac muscle results from the conformation space sampling. We estimate potential effects of conformation space sampling by using the tarantula myosin filament reconstruction (3jbh.pdb) as a model for relaxed cardiac muscle [27]. Single myosin measurements track lever-arm orientation relative to the thick filament axis using a specific region of the lever-arm helix equivalent to the black section of the human cardiac myosin lever arm in Fig 1 (residues 808–827, tarantula sequence) that we estimate for the tarantula myosin. Thick filament axis orientation is estimated using a specific region of S2 (residues 943–962, tarantula sequence). Thick filament axis orientation computed from each of the 4 myosins in the tarantula structure estimates a probability density for variability in thick filament structure that we assume single myosins sample during the course of the time-resolved lever-arm orientation measurement. We estimate the relaxed lever-arm step-size by computing Φ for lever-arm orientation relative to random variates of the thick filament structure substituting for a time-dependent trajectory. Φ’s are converted to step-size using Eq 3 and giving a step-size histogram comparable to observation.

## Results

### HCRLC-GFP stoichiometry in the zebrafish heart

**Fig 4:**
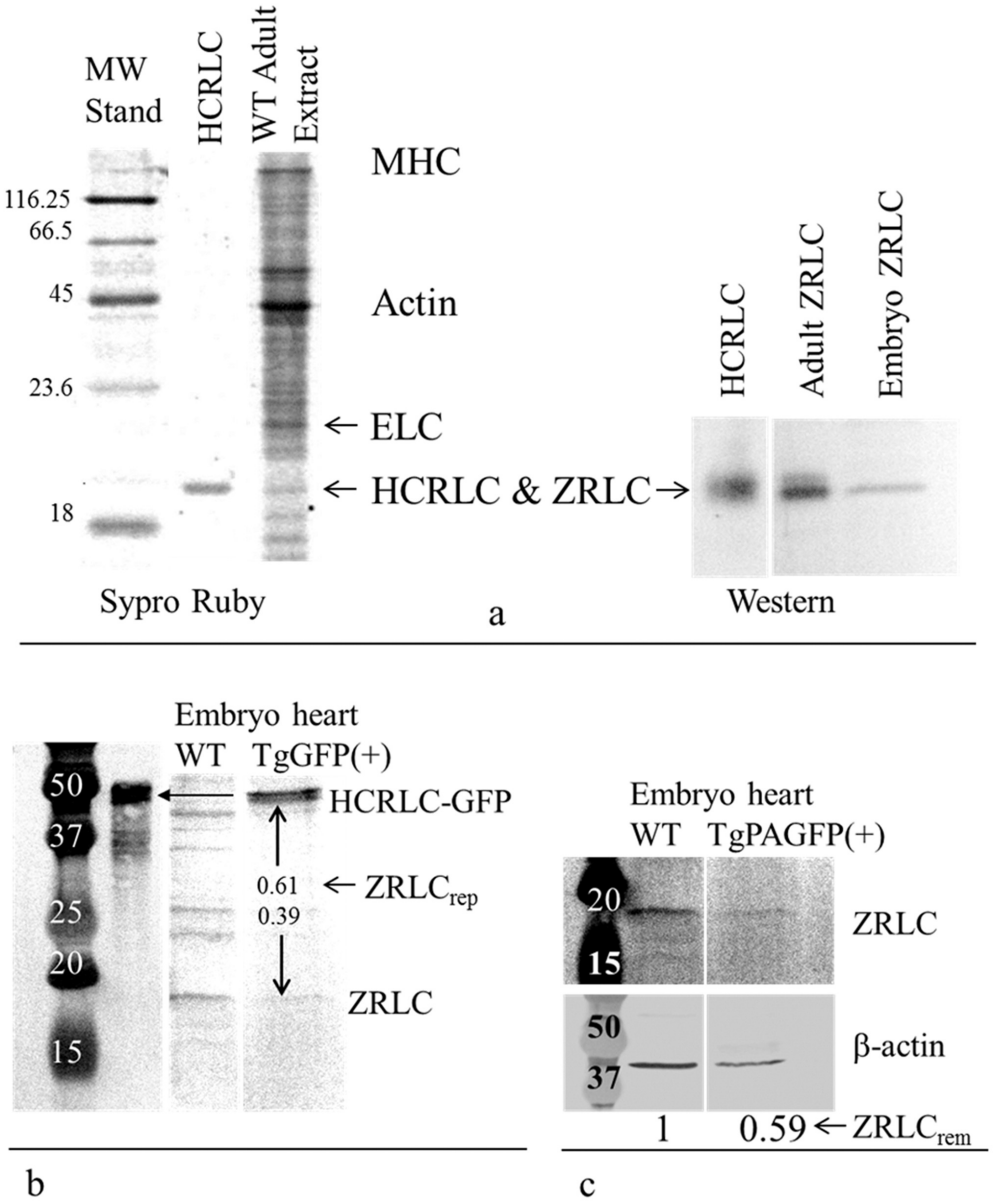
The Sypro Ruby stained and immunoblotted SDS-PAGE gels. The Sypro Ruby gel band intensities establish ZRLC content in a zebrafish adult heart extract relative to a known amount of in vitro expressed HCRLC (panel a). These samples produce calibrated blot intensities for HCRLC and ZRLC under a standardized protein immunoblotting protocol using the RLC antibody as described in Methods and previously [11]. The standards are compared to immunoblots from HCRLC-GFP and ZRLC in WT and TgGFP(+) embryos (**panel b**) to measure relative fractions of HCRLC-GFP and ZRLC content (ZRLC_rep_, Eq 1). The relative amount of ZRLC in WT and TgGFP(+) embryo hearts confirmed ZRLC content removed (ZRLC_rem_, Eq 2) by their comparison to control β-actin expression (**panel c**).

We detected the ZRLC and HCRLC-GFP protein content in WT and TgGFP(+) embryo and adult zebrafish hearts using SDS-PAGE and combining Sypro Ruby staining and immunoblotting for detection of protein content. Fig 4 shows the Sypro Ruby stained and immunoblotted SDS-PAGE gels. The Sypro Ruby gel band intensities establish ZRLC content in a zebrafish adult heart extract relative to a known amount of in vitro expressed HCRLC (**panel a**). These samples produce calibrated blot intensities for HCRLC and ZRLC under a standardized protein immunoblotting protocol using the RLC antibody as described in Methods and previously [11]. The standards are compared to immunoblots from HCRLC-GFP and ZRLC in WT and TgGFP(+) embryos (**panel b**) to measure relative fractions of HCRLC-GFP and ZRLC content (ZRLC_rep_, Eq 1). The relative amount of ZRLC in WT and TgGFP(+) embryo hearts confirmed ZRLC content removed (ZRLC_rem_, Eq 2) by their comparison to control β-actin expression (**panel c**) as described in Methods and previously [11]. Results agree that ZRLC_rep_ = 0.63 ± 0.04 where error is standard deviation for n = 6. Each sample size has ~80 embryos.

### Effect of HCRLC-GFP on zebrafish embryo heart contractility

**Fig 5:**
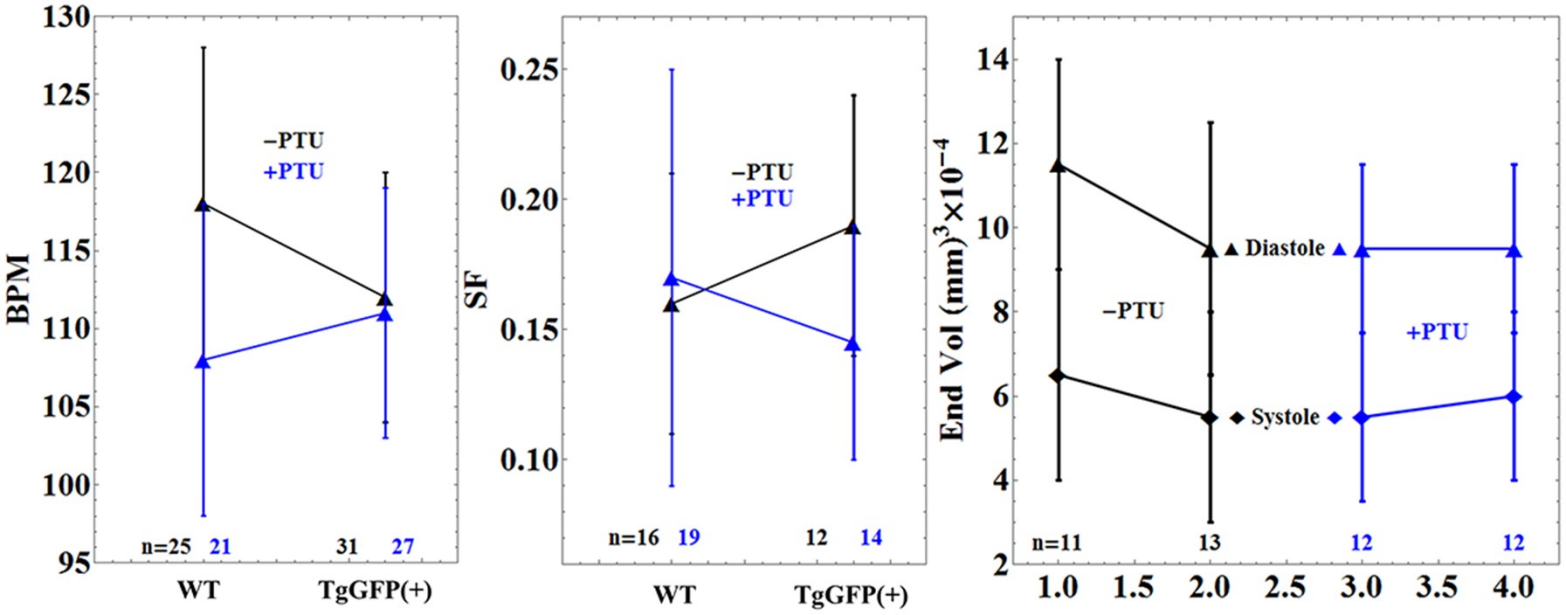
Comparison of heart rate in Beats Per Minute (BPM), Shortening Fraction (SF), and cyclical ventricle volume changes for WT and TgGFP(+) embryos in the presence (blue) and absence (black) of PTU treatment. Error bars show standard deviation for *n* embryos.

Fig 5 compares heart rate in beats per minute (BPM), shortening fraction (SF), and cyclical ventricle volume changes for WT and TgGFP(+) embryos in the presence (blue) and absence (black) of PTU. Error bars show standard deviation for *n* embryos. HCRLC-GFP incorporation into ~60% of the cardiac myosins in the heart has no significant impact on performance measured with these metrics. The PTU treatment inhibits melanogenesis and improves contrast in embryo heart images. It likewise has no significant impact on these metrics.

#### Quantitation of embryonic zebrafish cardiac myosin lever-arm swing during the heart beat cycle

**Fig 6:**
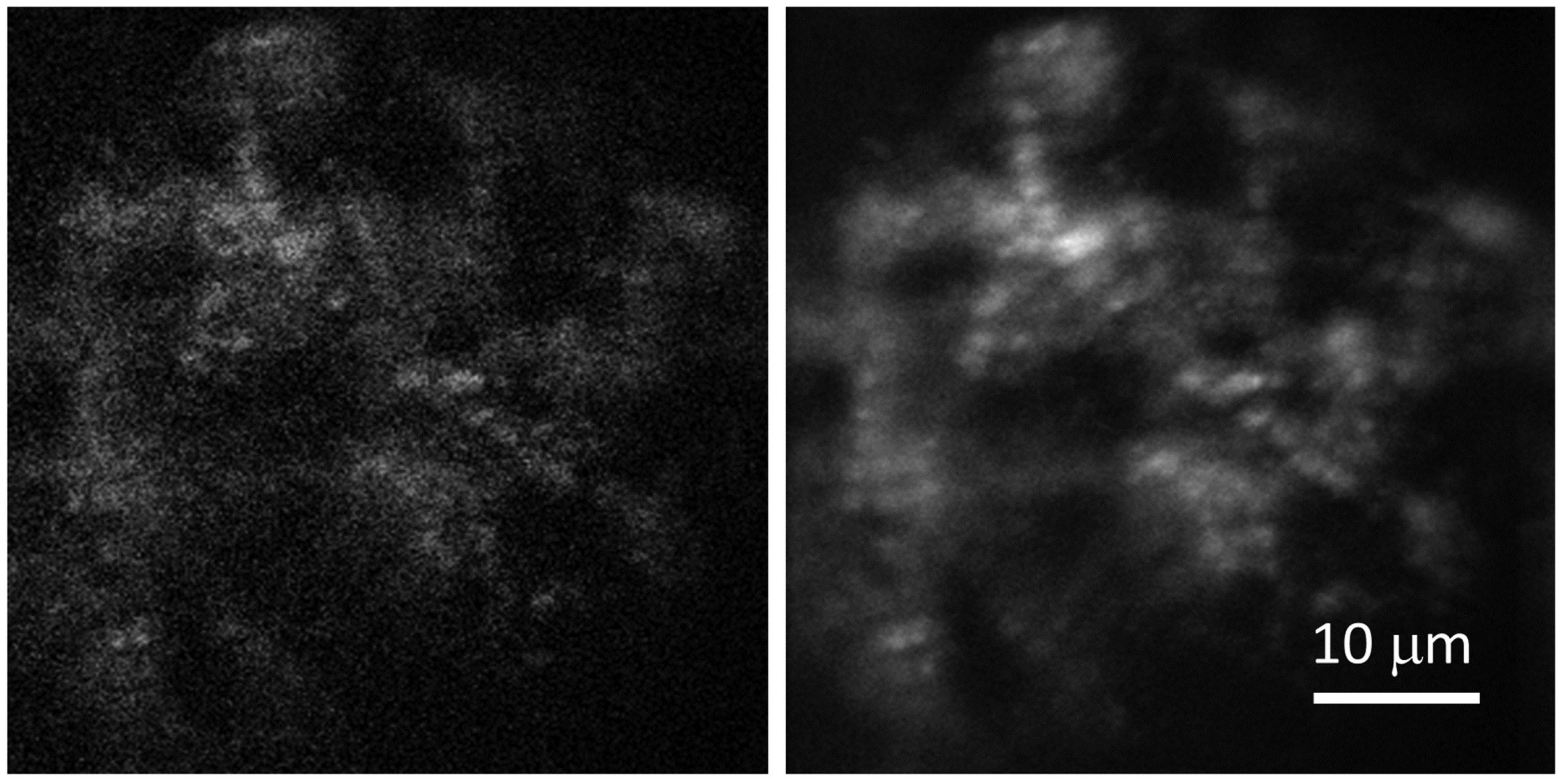
A single frame (left) and an averaged image of the heart (right) running at 50–60 bpm.

At 3–4 dpf, TgGFP(+) embryos were imaged to detect single cardiac myosin dynamics in the ventriculum as described in METHODS. Fig 6 shows a single frame from a movie and the averaged image of the beating heart where single molecule candidates are visible. This movie records ~6 sequential in-focus images of the sarcomeres followed by ~4 blurred images of shortening or otherwise moving sarcomeres and muscle fibers. In-focus images show approximately isovolumetric contraction since muscle shortening follows. We observed that the S/N ratio of the single myosin fluorescence is largest at highest isometric force just before ejection. We use this feature to synchronize the cardiac cycle with fluorescence intensity. The 1–2 highest S/N images were used for quantitation of the near-isometric contraction phase. The remaining 4–5 in-focus images were used for quantitation of the auxotonic contraction phase. We also stopped the heart by raising tricaine to 2200 mg/l in the microfluidic containing the embryo then briefly imaged the constantly-relaxed cardiac myosin.

Single myosin candidate images are isolated into 11x11 pixel movies from which the total intensity of each frame is plotted over time. From the intensity vs time plots we identify the single molecule photoactivation and photobleaching events thus qualifying the single myosin movie for inclusion into the data set. Figs A& B in S1 File indicate intensity vs time plots for single myosins in active and relaxed cardiac muscle.

The single molecule intensity patterns from zebrafish embryo single cardiac myosins were fitted using the pattern recognition algorithm and subjected to orientation super-resolution analysis [28, 29]. Time-resolved coordinates, having dipole or lever-arm helix spherical polar angles (β’,α’) or (β,α) defined relative to a fiber fixed frame (Fig 1), indicate the trajectories for a single dipole or lever-arm. Dipole orientation data impacts the best choice for the S1/GFP coordination. The new data representing cardiac myosin in relaxed and active muscle is consistent with the previous data selecting the S1/GFP coordination in Fig 1 over other docked models [10, 11]. The arc subtended by, Φ, the angle a single lever-arm helix rotates in sequential images defines a sequence of chords on a circle of diameter *L* equal to the lever-arm length indicated in Fig 3. Chord length is step-size, *d*, given by Eq 3.

We modeled the force developing homogeneous auxotonic and isometric phases, Δv_au_ and Δv_is_ using Eq 4 and as described there. Known relaxed and active step-size histograms v_re_, v_au_, and v_is_ are assigned their Poisson distributed noise and randomly sampled while ⌠v_au_ or Δv_is_ are minimized for each trial by selection of c_1_ and c_2_ or c_3_ and c_4_ subject to constraints c_1_, c_2_, c_3_, c_4_, ⌠v_au_, and Δv_is_ all ≥ 0 using constrained linear programing. We estimate average and variance for ⌠v_au_ and Δv_is_ from the trials. Standard deviation of the mean for ⌠v_au_ or Δv_is_ is computed for 15 or 24 cardiac muscle fibers from 8 or 6 embryos and corresponding to 1251 or 1436 single myosin coordinates from relaxed and active hearts, respectively.

**Fig 7:**
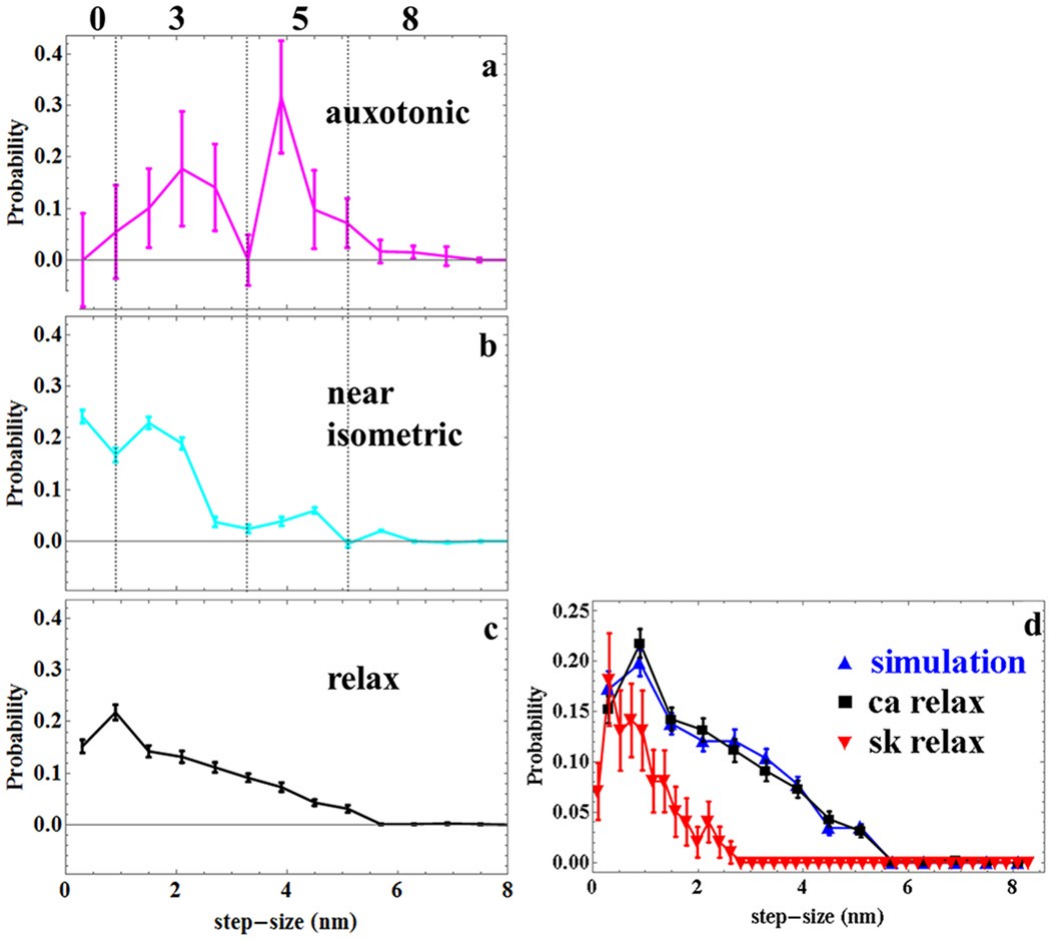
**Panels a-c. In vivo cardiac myosin active cycle from auxotonic (force developing), through near-isometric (maximum force), to detachment (relaxation) phases.** Error bars indicate standard deviation of the mean for 15 or 24 cardiac muscle fibers from 8 or 6 embryos and corresponding to 1251 or 1436 single photoactivated myosin coordinates from relaxed and active hearts, respectively. Dotted vertical lines in panels **a & b** define the boundaries between step-size frequencies computed from areas under the curves for nominal steps of 0, 3, 5, and 8 nm (nominal step-size corresponds to the in vivo measured step-sizes of 0, 2, 4, and 6 nm as indicated on the x-axis legend). Areas at step-size boundaries are split equally between adjoining step-sizes. **Panel d**. Simulated (blue) and observed (black and same as in **panel c**) relaxed cardiac (ca) myosin step-size distribution. Simulation is based on dispersion of thick filament structure surmised from the atomic model of Alamo et al. [27] as described in METHODS. In vivo relaxed skeletal (sk) myosin step-size from zebrafish embryo trunk muscle (red) is shown for comparison [31].

Fig 7 **panels a-c** display the cardiac myosin cycle from homogeneous auxotonic (⌠v_au_), through homogeneous near-isometric (⌠v_is_), to relaxation (v_re_). Fig 7 **panel d** compares in vivo skeletal myosin step-size in relaxation from zebrafish embryo trunk muscle [11] with the cardiac data. The skeletal (red) contrasts with the cardiac relaxed muscle (black) from the present study (**panels c or d**) where the relaxed cardiac myosins occupy a 2x larger step-size domain. The difference could be due to the higher time-resolution in the cardiac measurement or to other factors distinguishing cardiac and skeletal myosin. The former could convert a stationary relaxed lever-arm orientation observed from the in vivo skeletal myosin time-resolved trajectory into the non-stationary one observed for the in vivo cardiac myosin provided dynamic/static myosin rotational relaxation time was poised between 100 and 1000 ms. This is unlikely because rotation relaxation of the myosin head in relaxed skeletal muscle is on the order of 300–1000 ns [30]. Considering the latter, inter- and intra-myosin dimer interactions are known to impact relaxed myosin structure in cardiac thick filament [26] possibly leading to the more widely statically and dynamically distributed relaxed lever-arm [27]. We simulated the potential effect of thick filament interactions on relaxed cardiac myosin apparent step-size using the tarantula skeletal muscle thick filament structure as a model [27] and the approach described in METHODS. Fig 7 **panel d** compares simulated (blue) and observed relaxed myosin step-size distributions from cardiac (black and same as in **panel c**) and skeletal (red) zebrafish embryo muscle. Agreement between the cardiac muscle and simulated curves implies that inter- and intra-myosin dimer interactions impacting relaxed myosin structure in cardiac thick filament could explain the larger apparent step-size domain compared to the zebrafish skeletal muscle. While thick filament dispersion is likely also manifest in the skeletal muscle, our data suggests cardiac muscle myosin is dynamically distributed more widely on the 100 ms time scale.

Time-resolved single molecule experiments follow myosin through its cycle in real time. These data are summarized with event/step-size histograms also indicating step-frequency for the three unitary step-sizes observed. In vivo step-size from homogeneous auxotonic and near-isometric cardiac myosin in Fig 7 **panels a & b** indicates step-size probabilities peaking at ~2, 4 and 6 nm paralleling the unloaded in vitro estimates of ~3, 5, and 8 for zebrafish skeletal myosin [11]. Strain in the active cardiac myosin under load probably affects the apparent in vivo step-sizes by compacting the lever-arm rotation angle Φ. Fig 7 **panel b** indicates a force bearing 0 length step-size in the near-isometric phase that is unique to the in vivo myosin.

### Contraction cycle 4-pathway model

**Fig 8:**
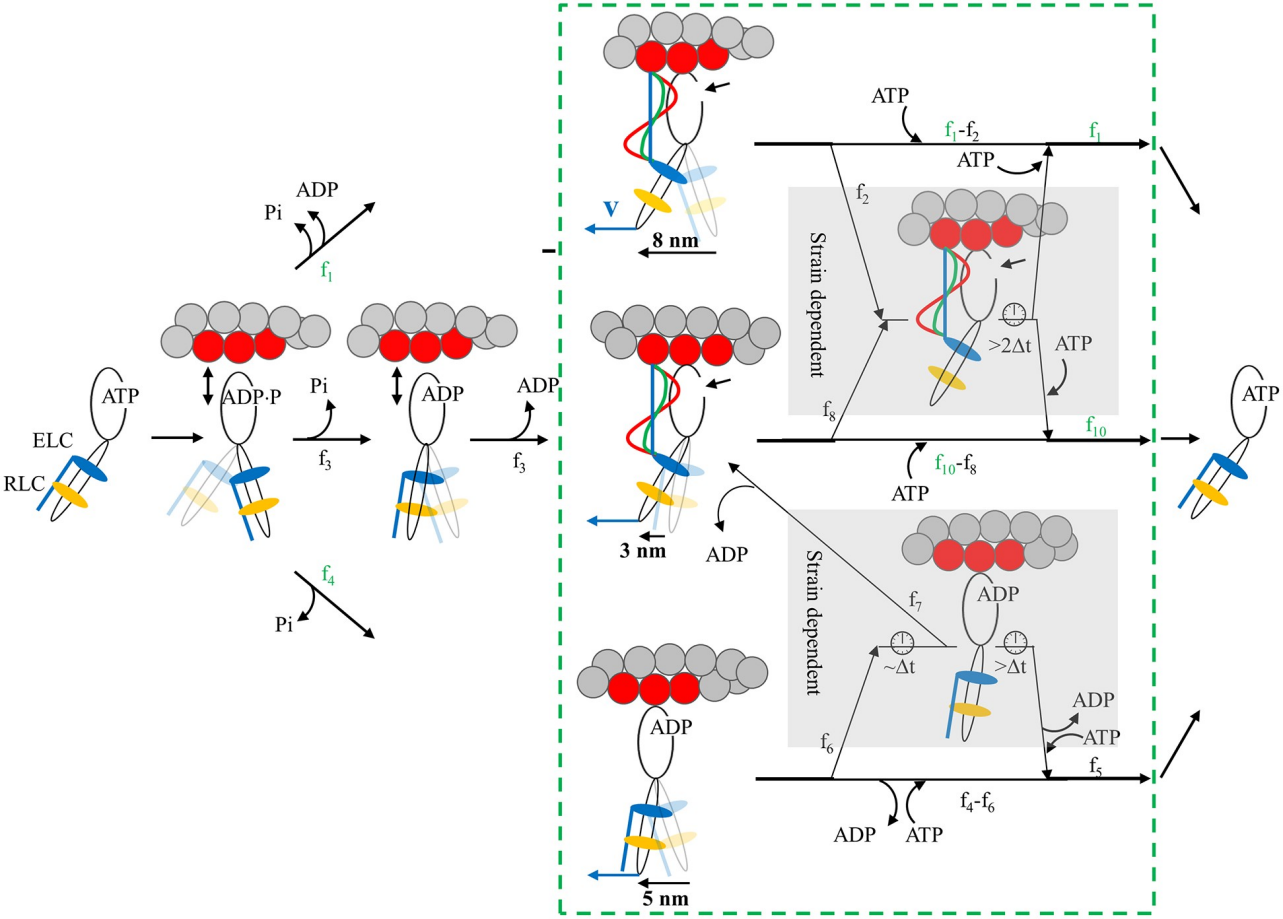
Myosin flux through the 4-pathway network contrasting 3 phases of muscle contraction in the beating heart. Myosin begins and ends detached from actin and with ATP bound in the contraction cycle. The green box with dashed line boundaries group the strong actomyosin bound states. Blue vector **v** at the end of the myosin lever-arm is positive net force on, and positive velocity of, the thick filament in units where amplitudes are equal. Fluxes through the network, f_i_, differ depending on contraction phases (values in Table 1). Measured values for f_i_ are in green while computed values are indicated in black. Four pathways cross from beginning to end of the contraction cycle. The top pathway populated by flux f_1_ executes an 8 nm step-size. The middle pathway populated by flux f_3_ executes a 3 nm step-size. It releases Pi while weakly actin bound without doing work. The bottom pathway populated by flux f_4_ is branched and executes 5 or 5+3 nm step-sizes. The branch from the bottom pathway is populated by flux f_7_ and executes the 5+3 nm step-size. Strain sensitivity is modeled with mechanisms in two subpathways within the shaded regions. The upper mechanism is populated by fluxes f_2_ and f_8_ from the 8 and 3 nm steps when the ELC N-terminus binds actin for actomyosin in rigor. The taut (blue line), intermediate (green curve), and slack (red wave) ELC N-terminus for muscle in near-isometric, auxotonic, or unloaded phases have high, modest, or zero strain when net force **v** is zero in isometric, intermediate in auxotonic, or high in unloaded phase. The linear (blue) actin bound ELC N-terminus is proposed to inhibit ATP binding by lowering active site accessibility for ATP at the small arrow near the myosin head. Inhibited ATP binding extends actomyosin attachment time indicated by the clock icon and quantitated in our single myosin measurements as a 0 length step. The lower mechanism is populated by flux f_6_ from the 5 nm step with ADP bound. Near-isometric, auxotonic, or unloaded phases have high, intermediate, or zero strain (of an unspecified myosin element) when net force is zero, intermediate, or high. Strain lowers ADP release rate. Short duration ADP rate inhibition flux, f_7_, leaves to continue with the 3 nm step. Long duration ADP rate inhibition flux, f_6_ − f_7_, continues with the 0 length step. For either the ATP accessibility or ADP release rate mediated mechanisms (top or bottom strain sensing mechanisms), low net force inhibits myosin cycling by extending the time myosin is strongly actin bound by >2Δt.

Fig 8 indicates a 4-pathway network producing 3, 5, and 8 nm steps (2, 4, and 6 nm in vivo) over the in vivo myosin cycle. Actin weakly attached states fall outside the dashed green box. Quantities f_i_ are myosin flux through the cycle with green and black indicating observed and computed values. We have shown previously using the Qdot assay that the basis of the 3, 5 and 8 nm unitary steps is the actin binding of the ELC N-terminus in a mechanism summarized in the figure [5]. Ignoring for a moment the shaded regions containing hypothetical strain dependent states, the bottom pathway with flux f_4_ performs the 5 nm step with release of product (Pi followed by ADP) but without attachment of the ELC N-terminus then ATP binding and actin detachment (f_5_). Alternatively, following a 5 nm step, slow ADP dissociation allows the ELC N-terminus to occasionally bind actin to make a subsequent 3 nm step with ADP release (f_7_) then ATP binding and actin detachment. The top pathway (f_1_) performs the 8 nm step with product release (Pi followed by ADP) and attachment of the ELC N-terminus then ATP binding and actin detachment. Pathways just described were identified previously in the context of the Qdot assay [5]. The middle pathway has flux f_3_ and a 3 nm step not preceded by a 5 nm step. It has Pi release without strong actin binding to avoid a 5 nm step, followed by ADP bound myosin strong actin attachment, then the 3 nm displacement. It is populated when actin has sufficient resisting force explaining its absence from the unloaded Qdot assay experiments. It is needed to accommodate the real situation when the quantity of 3 nm steps exceeds that of 5 nm steps. For each myosin state, blue vector **v** at the end of the myosin lever-arm is positive net force on, and positive velocity of, the thick filament in units where amplitudes are equal.

**Table 1:** ^a^. Cardiac myosin flux through *in vivo* active cycle in 3 phases.

phase	f_1_	f_2_	f_3_	f_4_	f_5_	f_6_	f_7_	f_8_	f_9_	f_10_
**Unloaded**	**39±7**	**0**	**5±8**	**56±9**	**43±11**	**13±9**	**13±9**	**0**	**0**	**18±12**
**Auxotonic**	**10±5**	**2±1**	**31±23**	**59±23**	**37±21**	**29±11**	**21±9**	**3±4**	**13±11**	**52±22**
**Near-isometric**	**2±1**	**1±1**	**80±5**	**18±5**	**12±3**	**15±4**	**6±2**	**42±5**	**52±4**	**86±3**
**notes**	**input & output**	**≤f**_**1**_	**input**	**input**	**output** **f**_**4**_**-f**_**7**_	**≤f**_**5**_	**5→3** **f**_**4**_**-f**_**5**_	**≤f**_**10**_	**f**_**2**_**+f**_**6**_**+f**_**8**_	**output** **f**_**3**_**+f**_**7**_

^a^ Flux quantities, f_i_, for the 4 step-size network defined in the Fig 8 model. Several flux values relate to known step-frequencies using Eq 5 and where f_1_ = x_8_, f_4_ = x_5_, f_9_ = x_0_, and f_10_ = x_3_. Other fluxes are surmised by using constraints. Flux conservation equality constraints include (total input) f_1_+f_3_+f_4_ = f_1_+f_5_+f_10_ (total output), f_4_ = f_5_+f_7_ (5 nm step input detaches or continues to 3 nm step), f_3_+f_7_ = f_10_ (3 nm step input sums with 5/3 nm step conversion then detaches with a 3 nm step), and f_2_+f_6_ −f_7_+f_8_ = f_9_ (total 0 length steps). The problem is under determined by equality constraints hence it is solved in two steps: first using equality constraints eliminating 4 parameters, second using 3 equality constraints relating f_2_, f_3_, f_5_, f_6_, f_7_, and f_8_ and inequality constraints for these variables. Inequality constraints are f_2_ ≤ x_8_, f_2_ ≤ x_0_, f_3_ ≥ x_3_-x_5_, f_3_ ≤ x_3_, f_5_ ≤ x_5,_ f_6_ ≤ x_5_, f_7_ ≤ f_6,_ f_7_ ≤ x_5,_ f_8_ ≤ x_3_, f_8_ ≤ x_0_, and all unknowns ≥ 0. The latter equality and inequality constraints are sufficiently restrictive to identify convergent solutions for the fluxes for each phase using constrained linear programing in Mathematica. We estimate standard deviations for fluxes within all constraints by generating random variates using normal distributions for {ω_0_, ω_3_, ω_5_, ω_8_} in Table 2, computing x_0_, x_3_, x_5_, and x_8_ using Eq 5, then solving for the unknown flux values. Flux errors are standard deviation for (n) replicates. Total input (or equivalently total output) is re-normalized to 100% post hoc facilitating comparison between phases.

**Table 2:** ^a^. Step probabilities.

phase	ω_0_	ω_3_	ω_5_	ω_8_
**Unloaded (n = 27)**	**0**	**0.13±0.04**	**0.50±0.09**	**0.37±0.08**
**Auxotonic (24)**	**0.03±0.12**	**0.44±0.27**	**0.45±0.17**	**0.08±0.04**
**Near-isometric (24)**	**0.33±0.02**	**0.54±0.04**	**0.12±0.03**	**0.01±0.01**

^a^ Step-probabilities measured from the data in Fig 7 panels a & b for 0, 3, 5, and 8 nm steps (0, 2, 4, and 6 nm in vivo) computed from areas under the probability curves and for auxotonic and isometric phases. Areas are defined in the figure panels by the vertical dotted lines with probability at the step-size boundaries split equally between adjoining areas. Data for the unloaded phase is taken from earlier in vitro work on porcine ventricular myosin (MYH7) [5, 17]. Step-probabilities {ω_0_, ω_3_, ω_5_, ω_8_} are normalized to sum to 1. Errors are standard deviation for (n) replicates.

Shaded regions in Fig 8 contain models for long lived strained conformation states in two mechanisms. The upper mechanism is populated by fluxes f_2_ and f_8_ from the 8 and 3 nm steps when the ELC N-terminus binds actin and the myosin is in rigor. The taut (blue) versus slack (red) ELC N-terminus in near-isometric versus unloaded phases have high versus zero strain when net force (or velocity) is zero in isometric contraction versus >> 0 in unloaded conditions. Taut ELC N-terminus is proposed to strain myosin in rigor (strained rigor) inhibiting ATP binding by lowering active site ATP accessibility as indicated by the size of the opening at the arrow near the myosin head. Delayed ATP binding and detachment (for >2Δt) gives a state observed as the force bearing 0 length step (f_2_ or f_8_). Slack ELC N-terminus has only prompt ATP binding and actin detachment (f_2_ = f_8_ = 0). The lower mechanism is populated by flux f_6_ from ADP bound myosins. Near-isometric versus unloaded phases have high versus zero strain when net force (or velocity) is zero in isometric contraction versus >> 0 in unloaded conditions. ADP release rate is diminished in the former case. Spontaneously slow ADP release that is not caused by strain allows additional time (~Δt) for the ELC N-terminus to bind actin and perform a 3 nm step. Flux f_7_ follows this pathway. The remainder has delayed ADP release caused by strain (for >Δt) giving a state observed as a 0 length step. Unloaded conditions without strain have f_6_ = 0 with prompt ATP binding and actin detachment (f_5_) or a subsequent 3 nm step (f_7_). For either mechanism, the net effect of high strain is to inhibit myosin cycling by extending the time by >2Δt that myosin is strongly actin bound giving the 0 length step state with a summed flux, f_9_ = f_2_ + f_6_ −f_7_ + f_8_, measured by the data in Fig 7.

The force bearing 0 length step-size involves a temporary diversion of myosin flux into an inhibited state that does not provide a parallel pathway though the myosin active cycle. Traditional normalization has step-frequencies for the 3, 5, and 8 nm steps summing to 1 such that,

$$x_{0}=\frac{\omega_{0}}{1-\omega_{0}},\;x_{3}=\frac{1-\left(\omega_{0}+\omega_{5}+\omega_{8}\right)}{1-\omega_{0}},\;x_{5}=\frac{\omega_{5}}{1-\omega_{0}},\;and\;x_{8}=\frac{\omega_{8}}{1-\omega_{0}}$$ (5)

where ω_0_, ω_3_, ω_5_, and ω_8_ are areas under the probability curves for the ~0, 3, 5, and 8 nm step-sizes indicated in Fig 7 **panels a & b**. Flux values for all phases studied are summarized in Table 1. The caption provides additional details about quantitation of ω_0_, ω_3_, ω_5_, and ω_8_ from Fig 7 and the optimization routine to obtain fluxes not directly observed.

Step-frequencies quantitate step-size probability but they are ambiguous in the 4-pathway network since a particular myosin passing through its cycle could do 5 then 3, 5, or 3 nm steps. The relative fluxes through the 4 pathways in Fig 8 are unique in this regard. Table 3 summarizes myosin 4-pathway flux for the beating zebrafish embryo heart. They accommodate the new step-size generating model in which the 3 nm step has a dedicated input pathway in addition to the 3 nm step dependent on input to the 5 nm step.

**Table 3:** Myosin flux through the 4-pathway network in a zebrafish embryo beating heart.

	step-size (in nm) ^a^ and normalized flux ^b^			
phase	8 nm	5 nm (only)	3 nm (only)	5 then 3 nm
Unloaded (n = 27) ^c^	39±7	43±11	5±21	13±9
Auxotonic (24)	11±5	43±22	25±31	21±9
Near-isometric (24)	2±1	12±3	80±6	6±2

^a^ Step-sizes in vivo are ~6, 4, and 2 nm.

^b^ Fluxes reflect total input or output at 100% for each phase. Errors are standard deviation for (n) replicates.

^c^ Data for the unloaded phase is taken from earlier in vitro work on porcine ventricular myosin (MYH7) [5, 17].

The unloaded phase in Fig 8 occupies the earliest part of the force producing cycle when velocity and net force are positive and largest. We model it with characteristics from the unloaded in vitro assay and under the assumption that there are no 0 length steps (f_9_ = 0). The ELC N-terminus is shown as a slack curve (red) indicating it does not undergo enhanced or prolonged strain for large velocity and net force (**v** >> 0). Input flux separates 39/5/56 into the 8, 3, and 5 nm step pathways (f_1_, f_3_, and f_4_) where f_1_ and f_4_ are derived initially from the traditional step-frequency percentages (ω_8_ and ω_5_) of 37 and 50% then undergo input normalization to accommodate unknown f_3_. A fraction of myosins completing the 5 nm step release ADP with the attachment of the ELC N-terminus (f_6_ = f_7_) to perform a subsequent 3 nm step. These are joined by the direct contribution (f_3_) together giving 18% of the output flux f_10_. The pure 5 nm step output (f_5_) contributes 43% and the 8 nm step (f_1_) independently contributes 39% of the output flux. Output flux is f_1_+f_5_+f_10_ = 100%.

The auxotonic phase in Fig 8 produces lower velocity and net force than the unloaded phase. It is characterized directly from the in vivo step-size data (Fig 7). The total 0 length step flux (f_9_) is approximately equivalent to error (Table 1). The observation that few 0 length steps occur in the auxotonic phase lends credibility to the assumption that the unloaded phase makes no 0 length steps. The ELC N-terminus is shown as a nearly taut curve (green) representing modestly enhanced or prolonged strain when velocity and net force are modestly positive (**v** > 0). Input flux separates 10/31/59 into the 8, 3, and 5 nm step pathways (f_1_, f_3_, and f_4_). The input flux of the 5 nm step (f_4_) is about equal to that in the unloaded phase but more of these myosins also undergo the coupled 3 nm step (f_7_). These are joined by the direct contribution (f_3_) together giving all of the output flux f_10_. Thus the 3 nm step-size flux enjoys a ~3-fold increase over the unloaded phase. The 8 nm step suffers a 4-fold reduction in flux from 39 to 10% (f_1_) when compared to the unloaded phase. The modest net force probably lowers ADP release rate sufficiently to shift myosin flux into f_7_ favoring the 3 nm step pathway over the terminal 5 nm step pathway (f_5_) but insufficiently to produce significant 0 length steps.

The near-isometric phase in Fig 8 produces near zero velocity and net force. It is also characterized directly from the in vivo step-size data (Fig 7). The 0 length step flux at 52% (f_9_) indicates that half of the cardiac myosins have inhibited detachment from actin due to strain. The ELC N-terminus is shown as a taut curve (blue) representing strongly enhanced or prolonged strain when velocity and net force are nearly zero (**v** ≈ 0). Input flux separates 2/80/18 into the 8, 3, and 5 nm step pathways (f_1_, f_3_, and f_4_). The terminal 5 nm step-size flux (f_5_) is low with 3 out of 4 myosins first undergoing strain dependent inhibition. The 3 nm step-size flux (f_10_) accounts for most myosins at 86%, a large increase over auxotonic phase, but in this case most myosins (80%) arrive there by the direct route with f_3_. The 8 nm step-size flux at 2% (f_1_) is negligible. The zero net force is proposed to enhance or prolong ELC N-terminus strain in strained rigor to inhibit ATP binding and strongly shift myosin flux (f_6_ and f_8_) to favor the 0 length step pathways. High flux through the 3 nm step-size pathway implies it clears the backlog of high free energy or tension producing myosins at near-isometric force as it can continue to move myosins through the active cycle when the other step-size pathways cannot indicating how the motor down shifts to smaller step-size pathways in the near-isometric phase.

Dominance of the 3 nm step in isometric contraction implies a deeper significance because this step results from actin binding of the ELC N-terminus. It implies the ELC N-terminus is the nanomotor ratchet that locks the myosin into a force bearing state (0 length step) until there is additional forward movement from another motor in the ensemble or when loading force decreases with the movement of blood such that the ratchet can release locked myosin to complete its ATPase cycle.

## Discussion

Myosin in striated muscle transduces ATP free energy into the mechanical work of moving actin. It does so using cyclical rotary movement of the lever-arm/light-chain complex linking motor generated torque to the myosin filament backbone. The linear actin displacement due to lever-arm rotation defines the unitary myosin step-size. The essential in vitro myosin structure/function paradigm is captured by its single molecule mechanical characteristics measured using the Qdot assay characterizing motor step-size and step-frequency [32]. Muscle myosin performance beyond the essential structure/function paradigm is influenced by self-assembly and integration with other motors and proteins in the muscle sarcomere. The native integrated myosin, with potential for hierarchical coordinated functionality and regulation because of its structured environment, was investigated here with cardiac myosin in live zebrafish. We developed tools to measure and interpret in vivo single cardiac myosin lever-arm rotation in a beating heart and estimate the cardiac myosin step-size and step-frequency. These metrics provide unprecedented insight into native cardiac myosin structure/function.

Myosin unitary step-size and rotation (tilt) has been studied for a long time. Ensemble myosin techniques used active muscle quick release or quick stretch length transients followed by time-resolved measurements of force [33], myosin polarized fluorescence [34–36], and X-ray diffraction [37] to compute the average unitary step-size or tilt under various loads. Later, because of the laser trap [38], in vitro single myosin step-size measurements became a standard and loaded myosin had a single, shorter, step-size [39]. Interest remained in ensemble force and X-ray methods because they worked on live excised tissue where average step-size was likewise shown to shorten with load. With transgenic zebrafish and the activatable tag on myosin, measuring in vivo single myosin step-size is now possible as we report here. Although our work on in vivo cardiac myosin extends our in vitro work where we identified the 3 unitary step-sizes and their step-frequencies [17], there is also overlap and general agreement with the ensemble average step-size measurements indicating a shorter average step with higher load. Comparing ensemble average and single myosin results we find there are three critical distinctions. We report that the average step-size shortens under increasing load due to shifting frequencies among short, intermediate, and long unitary step-sizes, we directly observe the force bearing 0 length step, and the mechanism for how all of it is accomplished involving ELC N-terminus actin binding follows directly from earlier in vitro single myosin measurements [5, 17]. The latter indicates that shifting step-frequencies to favor the short step is a downshifting maneuver caused by the ratcheting effect of the actin bound ELC N-terminus also showing that the in vitro and in vivo systems closely correlate.

Fig 7 **panel d** contrast in vivo relaxed cardiac and skeletal myosin apparent step-size probabilities indicating a more orientationally dynamically dispersed native cardiac myosin lever-arm. Cardiac myosin is known to maintain various special conformations in relaxation related to a super-relaxation state [26, 40–42] and the effect of RLC phosphorylation [43]. These circumstances could impact relaxed myosin conformational sampling. We modeled this effect using the structural heterogeneity indicated in an atomic model reconstruction for tarantula myosin filament [27] and estimated relaxed lever-arm apparent step-size by computing Φ for random variates of the thick filament structure substituting for a time-dependent trajectory. Φ’s are converted to step-size using Eq 3 giving a pseudotime-dependent single myosin signal that compares favorably to observation as indicated in Fig 7 **panel d**. While divergence of in vivo lever-arm time-dependent trajectories in cardiac and skeletal muscle could be from the higher time-resolution in the new cardiac measurement, earlier work with relaxed porcine cardiac ventricular myosin lever-arm orientation from permeabilized papillary muscle fibers indicated a similar pattern when compared to skeletal muscle at equivalent time resolutions [10]. We conclude that the divergence of in vivo cardiac and skeletal observations on relaxed muscle relates to the special conformations of cardiac thick filament.

Myosin translates actin when the two proteins are strongly bound. In the Qdot assay, unitary actomyosin interactions are characterized in vitro with super-resolution microscopy [17]. Qdot assaying of porcine β-ventricular myosin (βmys) indicated three unitary steps-sizes of ~3, 5, and 8 nm with relative step frequencies of ~13, 50, and 37% [17]. Similar results were obtained using the assay for adult zebrafish skeletal myosin step-size and step-frequency [11]. ELC N-terminus binding to actin is the mechanism for generating the 3 unitary steps [5] that is modeled in a 4-pathway network in Fig 8.

In vivo step-size and step-frequency from auxotonic and near-isometric cardiac myosin in Fig 7 **panels a & b** indicates step-sizes of ~2, 4 and 6 nm paralleling the unloaded in vitro estimates of ~3, 5, and 8 for porcine cardiac myosin plus a force producing 0 length step unique to the native in vivo environment. Strain in the active cardiac myosin under load probably lowers measured in vivo step-sizes by affecting the apparent lever-arm rotation angle Φ and also causing the 0 length step. The step-size and step-frequency data from Fig 7 **panels a & b** are interpreted in Fig 8 showing myosin flux through the 4-pathway network for unloaded, auxotonic, and near-isometric phases of cardiac muscle contraction. Increasing load (smaller net force or velocity **v**) changes step-frequencies to favor shorter steps such that in near-isometric conditions the long 8 nm step is practically eliminated, the 5 nm step is down-regulated from the unloaded value of 43 to 12%, while the shortest 3 nm step is up-regulated from the unloaded value of 5 to 80% of the total steps (Table 3). Uniquely, the 5 then 3 nm step up regulates then down regulates when the muscle transitions from unloaded to isometric phases suggesting its role is to better accommodate rapidly changing force production conditions when the 8 nm step is too long but the 3 nm step is too short to maintain power requirements. That the 3 nm exceeds 5 nm step-frequency under high load implied the new mechanism for step-size generation where the 3 nm step contributes both independently and together with the 5 nm step. This is unlike previous assertions that the 3 nm step strictly follows on the 5 nm step based on in vitro work [5]. Increasing load also causes the muscle to make use of the force producing 0 length step that holds tension by remaining strongly actin bound when sliding velocity is near zero. We find that the correlation of rising 0 length and 3 nm step-frequencies with lower 5 and 8 nm step-frequencies and higher resisting force implicates the ELC N-terminus and strained rigor conformation in strain sensing. This observation could be at odds with earlier in vitro motility work looking at strain-dependence in mouse cardiac myosin under load where strain enhanced ATP affinity for actomyosin [44] although loads were short of near-isometric where the ELC ratchet is expected to lower ATP affinity for actomyosin. The video file in S5 Movie has an audio/visual description/representation of myosin flux through the 4-pathway network during unloaded, auxotonic, and near-isometric contraction. The written description for the S5 Movie is in S2 File. The latter elaborates on the audio portion of the movie and contains one equation.

The proposed mechanisms for strain sensing in vivo, depicted in Fig 8, has the ELC ratchet causing the time extended strained rigor state by inhibiting ATP binding, and, the strain dependent ADP release causing the time extended ADP state by inhibiting ADP release. The ELC mediated mechanism accounts for most (42 of 52%) of the total flux into the force producing 0 length step in near-isometric phase. The inhibition efficiency of the strain dependent mechanisms, defined as the flux into the 0 length step divided by the total flux through the contributing pathway, suggests that the strain-dependent ADP release mechanism shuts down flux to the 0 length step pathway from the 5 nm step pathway. Instead the flux travels through the less inhibiting ELC ratchet mediated strain dependent mechanism.

## Conclusion

Cardiac ventriculum myosin transitioning from low to high force causes motor down-shifting to a 3 nm step-size accounting for >80% of all steps in the near-isometric contraction phase. We propose that strain delays ATP dissociation of actomyosin at isometric force using a mechanism involving the ratcheting effect of the actin bound ELC N-terminus. The significance of ELC N-terminus actin binding follows directly from earlier in vitro single myosin measurements [5, 17] showing close correlation between in vitro and in vivo systems. Enhanced occupation of the 0 length step hints at a mechanism for restrictive cardiomyopathy (RCM) caused by mutation in ELC [45]. Strongly bound myosins fail to detach quickly due to a fault in the ELC N-terminus strain sensing inhibiting detachment under low load conditions. It produces a drag force during stretch when the muscle should be fully relaxed.

We explored transduction/mechanical coupling in the beating heart at the single myosin level. High time resolution imaging captures and discriminates the native single myosin behavior during auxotonic and isometric phases of its contractile cycle providing deep insight into the significance of the cardiac myosin strain dependent biochemistry. Overall our approach provides a unique bottom-up assessment of the muscle motor performance by combining high precision single myosin mechanical characterization in an integrated and hierarchically functioning native muscle machine.

## Supporting information

S1 File S1 File consists of two figures (A &B) with captions. (DOCX)

S2 File S2 File contains a description of S5 Movie. (DOCX)

S1 Movie S1 Movies contains the video Data147bs(43,171,38,106,90)1. (AVI)

S2 Movie S2 Movie contains the video Data149bs(190,180,67,121,90)1. (AVI)

S3 Movie S3 Movie contains the video Data1bs(182,168,98,163,55)1. (AVI)

S4 Movie S4 Movie contains the video Data230bs(214,217,61,110,45)3. (AVI)

S5 Movie S5 Movies contains the video Movie5. (WMV)
