# Supplementary material for: Auxotonic to isometric contraction transitioning in a beating heart causes myosin step-size to down shift
Source: PLoS One. 2017 Apr 19;12(4):e0174690. doi: 10.1371/journal.pone.0174690 (PMC5396871; doi:10.1371/journal.pone.0174690)
Supplement: S1 File — (DOCX) [file pone.0174690.s001.docx]

Supporting File 1

Auxotonic to Isometric Contraction Transitioning in a Beating Heart Causes Myosin Step-Size to Down Shift

Thomas P. Burghardt^1,2,3^, Xiaojing Sun^1^, Yihua Wang^1^, and Katalin Ajtai^1^

November 2016

^1^ Department of Biochemistry and Molecular Biology and ^2^ Department of Physiology and Biomedical Engineering, Mayo Clinic Rochester, Rochester, MN 55905

^3^ To whom correspondence should be addressed. Email: burghardt@mayo.edu


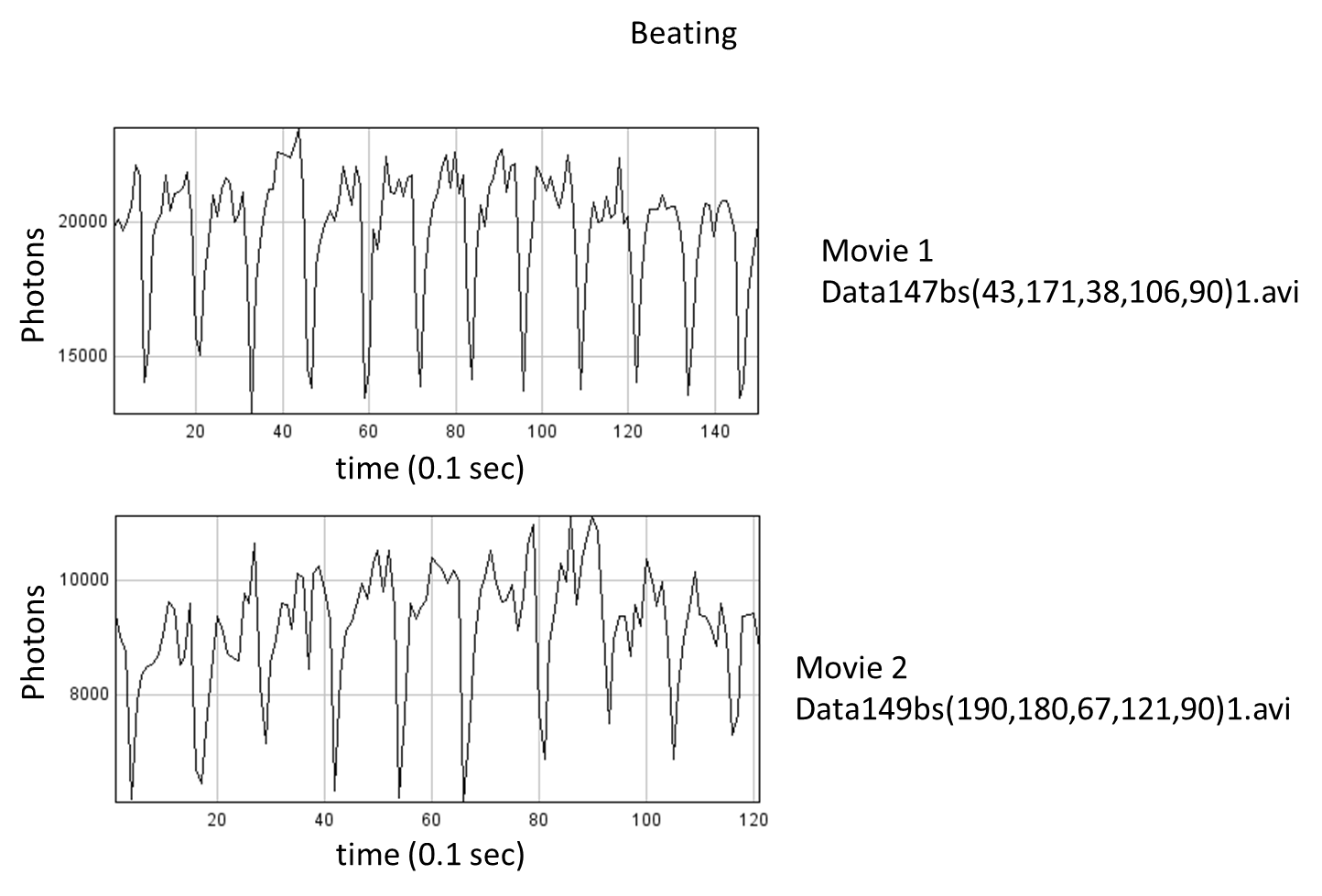


**Fig A**. Raw GFP fluorescence vs time from RLC-PAGFP tagged myosin *in vivo* from beating zebrafish embryo heart ventriculum under HILO illumination. It identifies single myosins by their quantized intensity change of ~1000 photons per 0.1 second above background due to photoactivation and subsequent photobleaching to background. Counts indicate intensity integrated over the EMCCD camera 11x11 pixel array containing a photoactivated chromophore. Accompanying video files, with name listed next to the fluorescence vs time plots, contain the raw images.


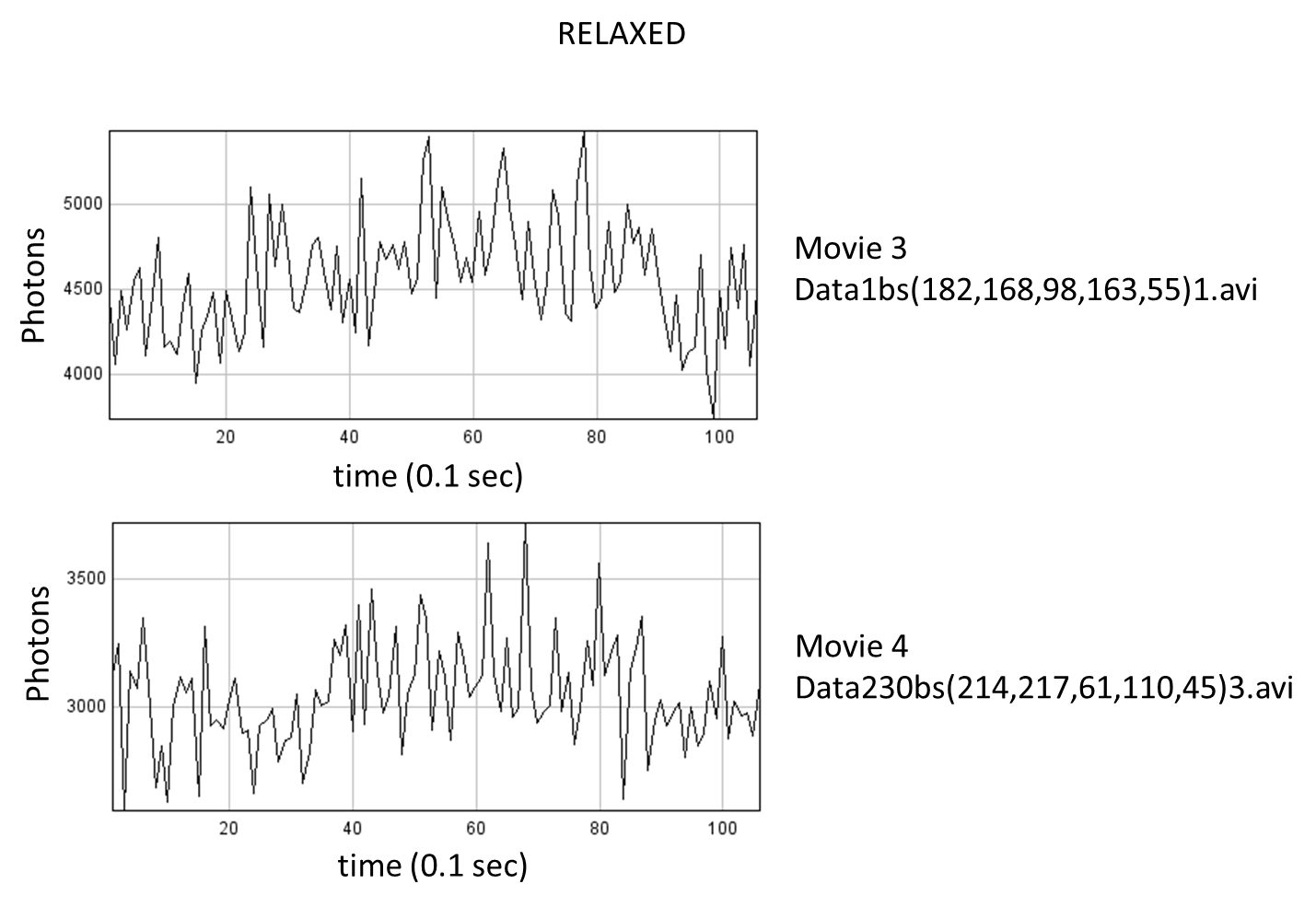


**Fig B**. Same as **Fig A** except for relaxed zebrafish embryo cardiac muscle. Accompanying video files, with name listed next to the fluorescence vs time plots, contain the raw images.
