# Supplementary material for: Auxotonic to isometric contraction transitioning in a beating heart causes myosin step-size to down shift
Source: PLoS One. 2017 Apr 19;12(4):e0174690. doi: 10.1371/journal.pone.0174690 (PMC5396871; doi:10.1371/journal.pone.0174690)
Supplement: S2 File — (DOCX) [file pone.0174690.s002.docx]

Supporting File 7

Auxotonic to Isometric Contraction Transitioning in a Beating Heart Causes Myosin Step-Size to Down Shift

Thomas P. Burghardt^1,2,3^, Xiaojing Sun^1^, Yihua Wang^1^, and Katalin Ajtai^1^

November 2016

^1^ Department of Biochemistry and Molecular Biology and ^2^ Department of Physiology and Biomedical Engineering, Mayo Clinic Rochester, Rochester, MN 55905

^3^ To whom correspondence should be addressed. Email: burghardt@mayo.edu

**Movie 5.** Myosin flux through the 4-pathway network in **Fig 8**.

Accompanying video file, Movie5.wmv, contains a simplified representation of the 4-pathway network in **Fig 8** during unloaded, auxotonic, and near-isometric contraction.

The first scene shows the **Fig 8** contraction cycle in simplified form with pathways in equivalent relative positions. Two strain inhibited transitions indicated by thunderbolts are the ATP binding (upper) and ADP release steps. The **Fig 8** cycle distinguishes strain inhibited myosins that we call 0 length step force producers from those that make the 3-8 nm steps with two sub-pathways. In this simplified cycle the inhibited transition rate accounts for both sub-pathways with descriptive terms None, LO, MED, or HI for no, low, intermediate, or high inhibition efficiency that is ranked by dividing flux to the 0 length step by the total flux for the contributing pathways. Then inhibition efficiency, σ, is defined numerically with,

** (S1)

for ATP binding or ADP release steps. Descriptive terms are assigned such that: σ = 0 is none, 0< σ ≤ 0.25 is low, 0.25 < σ < 0.75 is intermediate, and σ ≥ 0.75 is high.

The second scene indicates flow through four pathways for 8 nm (blue), 5 nm only (green, lower branch), 5 then 3 nm (green, upper branch), and 3 nm (red) steps. The branched pathway splits just before the strain inhibited transition.

The third, fourth, and final scenes identify flux through each pathway for unloaded, auxotonic, and isometric phases, respectively. Flux quantity is indicated by transparency of the grey shade except for the predominant pathway that is shown in red with appropriate transparency. Note the ELC ratchet at the strain inhibited intermediate. It changes from slack in unloaded phase meaning inhibition efficiency of 0, to gently curved in auxotonic phase where inhibition is low for ATP binding but intermediate for ADP release, to taut in isometric phase where inhibition is medium for ATP binding but high for ADP release.

Transparency in % is 100 minus flux in **Table 2** from the main text.
